# Inheritance and Fitness Cost of Laboratory-Selected Resistance to Cry1Ab in *Hyphantria cunea* Drury (Lepidoptera: Arctiidae)

**DOI:** 10.3390/insects15110861

**Published:** 2024-11-04

**Authors:** Laipan Liu, Wenjing Shen, Zhentao Ren, Zhixiang Fang, Li Zhang, Xin Yin, Qi Yu, Biao Liu

**Affiliations:** Key Laboratory on Biodiversity and Biosafety, Nanjing Institute of Environmental Sciences, MEE, Nanjing 210042, China; swjb1982@163.com (W.S.); rztkkk@163.com (Z.R.); zxfang23@126.com (Z.F.); huanghe70885@sina.com (L.Z.); njfu_shin@163.com (X.Y.); 15968821722@163.com (Q.Y.)

**Keywords:** *Bacillus thuringiensis*, Bt poplar, inheritance of resistance, fitness cost, resistance management

## Abstract

Transgenic poplars have been used to control quarantine pests worldwide, such as the fall webworm (*Hyphantria cunea*, FW). However, the studies on the resistance mechanism of FW to Cry toxins are limited. This study obtained an FW strain with 45-fold resistance to Cry1Ab toxin by continuous screening in the laboratory. The resistance to Cry1Ab was autosomally completely recessive, and it had approximately 40-fold cross-resistance to Cry1Ac but no cross-resistance to Cry2Ab toxin. Analysis of indoor population life tables for the resistant and susceptible strains found no significant difference in the net reproduction rate (R_0_) between the two strains. The resistant strain had an insignificantly longer larval development duration and a comparable pupation rate, pupal weight, emergence rate, eggs laidper female, and egg hatching rate compared to the susceptible strain. This indicated that there was no fitness cost for Cry1Ab resistance. Our study helps in evaluating the risk of resistance to Cry1Ab toxin and the mechanisms of resistance to Cry1Ab toxin in FW.

## Simple Summary

As an invasive pest, the fall webworm has caused huge economic losses in agricultural and forestry production, and the development of resistance in the fall webworm to Cry1Ab toxin is an important issue in plant protection. Our study shows that there is no fitness cost for the resistance of the fall webworm to Cry1A toxin, which comes from autosomal recessive inheritance and does not lead to cross-resistance to Cry2Ab toxin. According to the results of this study, transgenic poplars expressing multiple insecticidal proteins should be used directly and shelters should be established to control the fall webworm more effectively and delay the development of resistance.

## 1. Introduction

The fall webworm *Hyphantria cunea* (FW) is widespread worldwide and has over 600 host plant species. It has become a major pest in Europe and Asia, causing significant economic losses to agriculture and forestry. It is mainly controlled by artificial physical, chemical, and biological means [1] that are time-consuming and have little effect. Since 2002, China has commercially promoted transgenic poplar trees expressing Cry1A toxin to control the fall webworm; this is the earliest example of forest insect-resistant genetic engineering [2]. The cultivation of insect-resistant transgenic plants can effectively control pests, although the resistance of target pests to Bt toxin is the biggest challenge for the long-term and safe cultivation of transgenic Bt crops [3].

Resistance screening in target pests is generally conducted in the laboratory to understand the characteristics of potential resistance genes, and preventive resistance management strategies are designed based on the results obtained from indoor strains to delay the development of Bt resistance in pest field populations to proactively address the problem of pest resistance to Bt crops [4]. Currently, refuge is a widely used Bt crop resistance management strategy [4,5]. This strategy requires the planting of a certain proportion of non-Bt host crops near Bt crops as a refuge for pest-susceptible populations. Refuges provide sufficient numbers of susceptible pests to cross with the few resistant individuals surviving on Bt crops. If Bt resistance is recessive, then heterozygotes are killed by the Bt crops. Therefore, the recessive inheritance of field resistance genes is an important condition for the successful implementation of this strategy. Sufficient refuges in the field, a low initial frequency of the Bt resistance gene, recessive resistance, the fitness cost associated with resistance, and incomplete resistance are beneficial in slowing the rate of evolution resistance in the field [6,7,8], and the fitness cost of evolved resistance can effectively delay the development of resistance [9].

Research on Bt resistance in target pests has mainly focused on traditional agricultural pests, and the studies on the resistance mechanism of FW to Cry toxins are limited. With the development of transgenic trees, it is necessary to strengthen the research on Bt resistance in forestry insects. In this study, Cry1Ab toxin was used to screen susceptible populations of FW to obtain Cry1Ab-resistant strains. The risk of resistance to Cry1Ab toxin in FW was evaluated by detecting the genetic mode, cross-resistance, and fitness of resistant strains of FW, with the aim of exploring the mechanism of resistance to Cry1Ab toxin and delaying the development of resistance in transgenic poplars.

## 2. Materials and Methods

### 2.1. Insects and Toxins

The susceptible strain RQ was collected from Renqiu, Hebei Province, in 2019 and had not been exposed to any toxins or pesticides since it was raised indoors.

Cry1Ab-resistant strain RQ1Ab: The RQ strain was screened for Cry1Ab toxin, and the surviving larvae were kept on non-toxin-exposed feed until pupation to reproduce and continue the screened strain; 8 successive generations were screened, with an average of 1440 newly hatched larvae screened in each generation. The concentration of Cry1Ab toxin was controlled at between 60 and 80% of the population mortality [10]. After the bioassay experiment was completed, the surviving larvae were selected and fed non-toxin-exposed feed until pupation to reproduce the continued RQ1Ab strain.

Artificial FW feed was purchased from the Chinese Academy of Forestry Sciences. The feeding conditions were 27 ± 1 °C, 75 ± 10% humidity, and 16 h light/8 h dark.

The dry-powder Cry toxin used in this experiment was purchased from Shanghai Youlong Biotechnology Co., Ltd., Shanghai, China and stored in a refrigerator at −80 °C. The toxin was dissolved using a 50 mM Na_2_CO_3_ buffer (pH 12.1, containing 5 mM EDTA and 10 mM EGTA) prior to bioassay.

### 2.2. Bioassay

We used a 5 mL pipette to deliver 900 μL of warm artificial feed to the bottom of each well in a 24-well culture plate, ensuring a level liquid surface without adhesion to the walls or apertures. After natural solidification at room temperature, the plates were stored at 4 °C before use. The Bt toxin was separately diluted into seven concentrations using 0.01 M phosphate-buffered saline PBS (pH 7.4). Prior to treatment, the plates were cooled below 30 °C, and each well was treated with 100 μL of the toxin–PBS mixture or a PBS control without toxin. Each concentration was added to 2 24-well plates. After air drying at room temperature, the plates were stored until further use. A newly hatched larva was introduced into each well of a 24-well culture plate and covered with two layers of nylon gauze to prevent escape. The plate was incubated in a chamber with constant temperature and light (27 ± 1 °C, 75 ± 10% humidity, and 16 h of light and 8 h of darkness daily) for 5 days. Dead larvae or those unable to grow or develop normally (weighing less than 5 mg) were recorded.

First-instar larvae were selected for Bt toxicity assays as Bt protein is effective against the neonatal larvae of FW. However, hatched larvae are usually more fragile, and handling them can lead to greater errors in larval size as larvae exhibit webbing behavior. Therefore, first-instar larvae (three days after hatching) were used in this experiment.

Toxin concentrations (in μg Cry1Ab per cm^2^ diet) were 0, 0.0625, 0.125, 0.25, 0.5, 1.0, 2.0, 4.0, and 8.0 for unselected strains (RQ). Toxin concentrations for RQ1Ab evaluated after 8 generations of selection of RQ were 0, 2, 4, 8, 16, 32, 64, and 128. Toxin concentrations for the F_1_ progeny from crosses between RQ and RQ1Ab were 0.125, 0.25, 0.5, 1.0, 2.0, 4.0, 8.0, and 16.0. The concentrations of toxins in μg Cry1Ab per cm^2^ diet screened for 8 generations of RQ1Ab were 2, 4, 8, 16, 32, 32, 64, and 64.

### 2.3. Assessing the Inheritance of Resistance

The male and female RQ1Ab moths were individually crossed with the unmated male and female RQ moths, and vice versa. The F_1_ hybrid offspring were backcrossed with RQ to obtain a sample size exceeding 50 because resistance to Cry1Ab exhibits incomplete recessiveness (refer to the section below). Biological assays were performed on RQ1Ab, RQ, reciprocal F_1_ hybrids, and backcrossed offspring to evaluate their sensitivities to Cry1Ab.

The dominance parameter, h, was calculated according to the following formula based on the survival rate for the diagnostic dose of Cry1Ab:

h = (F_1_ Survival rate − RQ survival rate)/(RQ1Ab Survival rate − RQ survival rate),

where h = 0 represents complete recessiveness and h = 1 represents complete dominance [11].

The dominance parameter D was calculated based on the LC_50_ values of RQ1Ab, RQ, and hybrid F_1_ generation [12]. D value = (2lg (LC_50_F_1_) − lg (LC_50_RQ) − lg (LC_50_RQ1Ab))/(lg (LC_50_RQ1Ab) − lg (LC_50_RQ)), D = −1 indicates complete recessiveness, while D = 1 indicates complete dominance.

### 2.4. Establishing a Life Table and Observing the Physiological Parameters of FW

Within a 12 h time frame from hatching, 270 randomly selected first-instar larvae of the RQ and RQ1Ab strains and F_1_ progeny (RS) were divided into 3 groups. The animals were fed an artificial diet. The larval duration and survival rate were observed and recorded. Within 24 h of pupation, the pupae were weighed, and their sex was identified. The duration and rate of adult emergence were also recorded. Female and male adults from the RQ and RQ1Ab populations were randomly paired in a 1:2 ratio. The number of eggs laid by individual females in successfully mated pairs was recorded (10 pairs per group, totaling 30 pairs). The hatching rate of the eggs was determined based on the number of successfully hatched *Lymantria dispar* larvae. The biological characteristics and relative fitness parameters of each population were calculated.

### 2.5. Calculating the Net Reproductive Rate R_0_ and Relative Fitness

Net reproductive rate: R_0_ = N_t+1_/N_t_, where N_t_ represents the initial number (first-instar larvae) of the American white moth population and N_t+1_ represents the number of first-instar larvae in the population after one generation of reproduction.

Relative fitness = R_0_ of resistant population/R_0_ of susceptible population.

### 2.6. Data Analysis

PoloPlus 2002 software (LeOra Software^®^ Inc., Berkeley, CA, USA) was used to calculate the concentration that causes 50% larval mortality, including the LC_50_ and its confidence limits, the slope of the concentration–response curve, and its standard deviation to determine the toxicity of different toxins in various strains. There was a significant difference between these two values if the 95% confidence limits of the two LC_50_ values did not overlap [13].

Furthermore, IBM SPSS Statistics 20 software was utilized to perform analysis of variance (independent sample *t*-test, α = 0.05) to compare the disparities in physiological parameters, intrinsic growth rates, and other indicators between the resistant and susceptible strains and their F_1_ progeny.

## 3. Results

### 3.1. Inheritance of Resistance

The RQ1Ab strain showed 45-fold resistance after eight generations of selection compared to the Cry1Ab-Susceptible strain (Table 1). The LC_50_ and slope values were quite similar between the resistant and susceptible strains in the F_1_ hybrid populations (Table 2), indicating that resistance to Cry1Ab toxin was autosomally inherited and not influenced by maternal effects. The F_1_ hybrid populations exhibited 1.5- and 1.3-fold resistance to Cry1Ab in reciprocal crosses, similar to the susceptible strain. By calculating the dominance, we found that the D value was close to −1 and the h value was equal to 0 (Table 2), suggesting that the resistance of the RQ1Ab strain to Cry1Ab was nearly completely recessive in its inheritance.

### 3.2. Cross Resistance Spectrum in RQ1Ab Strain

After eight generations of continuous selection, RQ1Ab developed 45-fold resistance to Cry1Ab and 40-fold cross-resistance to Cry1Ac. The LC_50_ values for Cry2Ab toxin in the RQ1Ab strain overlapped with those in the susceptible RQ strain, indicating no cross-resistance to Cry2Ab toxin (Table 3).

### 3.3. Fitness of the RQ1Ab Strain

**Figure 1:**
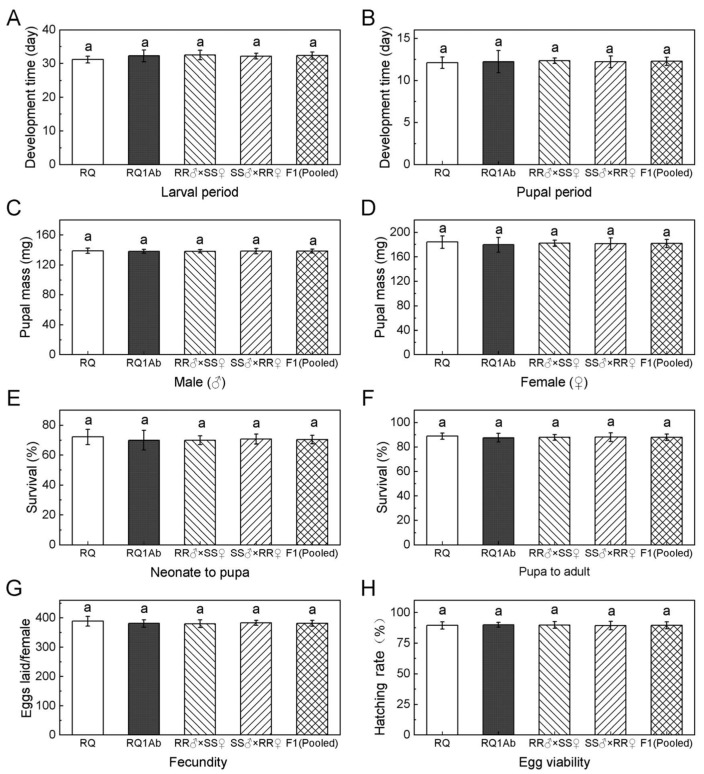
Comparison of fitness components among the susceptible strain RQ (SS), the Cry1Ab-resistant strain RQ1Ab (RR), and their F_1_ progeny (RR × SS) in *H. cunea* reared on artificial feed. The same lowercase letter in each image mean that the index does not differ significantly between different strains.

The resistant strain RQ1Ab and the F_1_ progeny exhibited a slightly longer developmental period, but showed no significant differences in the survival rate, egg production, and egg hatching rate compared with the susceptible strain RQ (Figure 1). The weight of female pupae in FW was notably higher than that of male pupae (*p* < 0.05) (Figure 1), whereas the weight of pupae of the same gender did not significantly differ between the resistant and susceptible strains (Table 4). Further analysis through life table construction for the RQ1Ab and RQ strains and their F_1_ progeny allowed for the determination of the net reproductive rate (R_0_) of these two strains (Table 4). RQ1Ab showed a relative fitness of 0.95 compared with RQ, which was not significantly different from that of RQ. This indicated that there was no fitness cost associated with resistance to Cry1Ab toxin in the RQ1Ab strain.

## 4. Discussion

Understanding the genetic basis of Bt toxin resistance in crop pest insects is important in evaluating the risk of resistance and designing effective resistance management strategies. In this study, the resistance of the RQ1Ab strain to Cry1Ab increased by 45 times after eight generations of selection. The resistance showed autosomal and completely recessive inheritance (h = 0) for the diagnostic dose of Cry1Ab. Resistance to Cry1Ab resulted in a 40-fold cross-resistance to Cry1Ac and no cross-resistance to Cry2Ab. Resistance to Cry1Ab did not appear to be associated with any fitness cost in the resistant strain, and the net reproductive rate did not significantly change.

Currently, high-dose refuge strategies represent the primary approach to delaying the development of resistance in target pests of Bt crops and are mandatory in countries such as Australia and the United States [14,15]. The refuge strategy is expected to ensure the survival of many susceptible individuals that can mate with a small number of surviving individuals in Bt crops. By integrating genetic theory with field practices, refuge strategies have the potential to effectively delay the evolutionary development of target insect resistance to Bt traits when the following conditions are met: (1) adequate refuge, (2) a low initial frequency of resistance genes, (3) recessive resistance, (4) the fitness cost associated with resistance, (5) incomplete resistance, and redundant killing with additional modes of action further delaying resistance [5,6]. The high-dose refuge strategy assumes that field resistance is inherited as a recessive trait. However, the results of laboratory screening for Bt Cry toxins indicate a distribution of resistance traits from complete recessiveness to complete dominance [16,17,18]. When resistance is recessive, the offspring produced by the mating of resistant and susceptible insects are unable to survive in high-dose Bt crops, significantly slowing the evolution of resistance [8]. Modeling results indicate that refuges of ≥5% can delay resistance development for over 20 years if resistance is completely recessive (h = 0) if the other parameter requirements are met. Furthermore, if the proportion of refuge is ≥50%, the development of resistance can be delayed by more than 50 years if all other parameters remain the same [4]. In this study, the resistance demonstrated autosomal and fully recessive inheritance (h = 0) for the diagnostic dose of Cry1Ab. This suggests that a reduced amount of refuge is required to effectively postpone the development of resistance in FW against transgenic poplar trees.

Gene pyramiding is one solution to overcome the issues of a narrow insect spectrum and the potential development of resistance in single-toxin Bt crops. The absence of cross-resistance between the expressed toxins is one of the conditions for the effectiveness of this strategy [7]. In the case of Cry1Ac and Cry2Ab, it is unlikely that these two toxins exhibit cross-resistance owing to the relatively low amino acid similarity in domain II between them [6,19]. However, some target pests of Bt crops have developed low but significantly different levels of cross-resistance to Cry1Ac and Cry2Ab, likely due to sequential exposure, first to Cry1Ac and then to Cry2Ab, including *Helicoverpa armigera* and its closely related species, *Helicoverpa zea* [6,10,19,20]. However, Cry1Ac selection in *H. armigera* populations from northern China also resulted in low-level cross-resistance to Cry2Ab toxin [21]. The results of the present study suggest that resistance to Cry1Ab did not induce cross-resistance to Cry2Ab, suggesting that cultivating *cry1ab*+*cry2ab* gene-stacked transgenic poplars in China is a favorable option.

The refuge provided by non-Bt host plants in the vicinity of crops with insecticidal traits plays a pivotal role in slowing the evolution of resistance to Bt toxins in pests. Resistance can lead to a lower fitness in resistant individuals compared to susceptible individuals in refuges, thereby increasing the refuges’ ability to delay the development of resistance [22]. Resistance emergence can delay the emergence of resistance when antagonistic or incomplete resistance is accompanied by a fitness cost [7,9]. Adaptation costs can be identified from the life history parameters of resistant insects (such as survival rate, growth rate, size, or reproductive capabilities) compared to those of susceptible insects. Additionally, adaptation costs can become apparent when resistance levels decline over time without selection pressure [23,24,25]. Therefore, accurately estimating the fitness costs is crucial in comprehending the evolution of resistance and determining effective resistance management strategies to mitigate resistance in genetically modified plants.

This study found no significant difference in the net reproductive rate (R_0_) between resistant and susceptible strains by constructing indoor population life tables. The resistant strain showed a slightly prolonged larval development period compared with the susceptible strain, although the difference was insignificant. There were no significant differences in pupation rate, pupal weight, emergence rate, female oviposition, or egg hatching rate between the resistant and susceptible strains, indicating that Cry1Ab resistance had no significant impact on the fitness of FW. The fitness of the resistant strain did not show a consistent performance in the absence of selection pressure [26,27,28,29]. Although the resistant strain selected in this study was recessively inherited, the absence of fitness costs may favor its continuous presence in refuges, potentially maintaining the frequency of resistant individuals in the field and facilitating the rapid development of resistance to Bt plants. Fitness is influenced by environmental conditions, such as diet, which means that costs in the laboratory and field may differ. However, fitness profiles found in laboratory studies should be considered when assessing the risk of evolving resistance in the field [28]. We recommend directly employing genetically engineered poplar trees with stacked genes expressing multiple insecticidal proteins and considering the genetic traits of the RQ1Ab strain to promote the use of genetically modified poplar trees in China. This strategy would result in enhanced control of FW and the postponement of resistance development. Future studies are required to determine the effect of using Cry1Ab toxin on the potential for resistance in other forest insects that feed on poplars and are killed by Cry1Ab.

## Figures and Tables

**Table 1:** Selection of resistance to Cry1Ab toxin in the RQ1Ab strain of *H. cunea*.

Generation	LC_50_ (95% FL) ^a^ (µg/cm^2^)	Slope ± SE	RR ^b^
0	0.48 (0.37–0.60)	1.53 ± 0.14	/
2	2.56 (2.00–3.26)	1.62 ± 0.17	5.3
4	6.56 (5.06–8.63)	1.48 ± 0.17	13.7
6	17.83 (12.51–24.98)	1.70 ±0.18	37.1
8	21.62 (15.56–30.37)	1.66 ± 0.17	45.0

^a^ Concentration of Bt toxins killing 50% of the larvae and their 95% fiducial limits. ^b^ Resistance ratio (RR): LC_50_ (RQ1Ab)/LC_50_ (RQ, 0.48 μg/cm^2^). There was a significant difference if the 95% confidence limits of the LC_50_ values did not overlap.

**Table 2:** Responses of the resistant strain (RQ1Ab), susceptible strain (RQ), and F_1_ progeny (RQ1Ab × RQ) of *H. cunea* to Cry1Ab.

Population	LC_50_ (95% FL) (µg/cm^2^)	Slope ± SE	*n*	RR	Dominance	
					D Value ^a^	h ^b^
Strain						
RQ	0.48 (0.37–0.60)	1.53 ± 0.14	432	1		
RQ1Ab	21.62 (15.56–30.37)	1.66 ± 0.17	384	45		
F_1_						
RQ1Ab♂ × RQ ♀	0.70 (0.57–0.87)	1.62 ± 0.13	432	1.5	−0.80	0
RQ♂ × RQ1Ab ♀	0.61 (0.49–0.77)	1.63 ± 0.14	432	1.3	−0.87	0

^a^ D value = (2lg (LC_50_F_1_) − lg (LC_50_RQ) − lg (LC_50_RQ1Ab))/(lg (LC_50_RQ1Ab) − lg (LC_50_RQ)), D = −1 indicates complete recessiveness and D = 1 indicates complete dominance. ^b^ h = (F_1_ survival rate − RQ survival rate)/(RQ1Ab survival rate − RQ survival rate); survival rate means survival rate at the diagnostic dose of Cry1Ab; h = 0 represents complete recessiveness; and h = 1 represents complete dominance. There was a significant difference if the 95% confidence limits of the LC50 values did not overlap.

**Table 3:** Responses of RQ1Ab and RQ strains to Bt toxins.

Strain	Toxin	LC_50_ (95% FL) (μg/cm^2^)	Slope ± SE	Number	RR ^a^
RQ1Ab (Selected)	Cry1Ab	21.62 (15.56–30.37)	1.66 ± 0.17	384	45
	Cry1Ac	26.40 (20.95–33.53)	1.71 ± 0.18	384	40
	Cry2Ab	0.95 (0.76–1.19)	1.71 ± 0.16	432	1
RQ (CK)	Cry1Ab	0.48 (0.37–0.60)	1.53 ± 0.14	432	
	Cry1Ac	0.66 (0.51–0.85)	1.41 ± 0.14	432	
	Cry2Ab	0.81 (0.62–1.07)	1.29 ± 0.13	432	

^a^ Resistance ratio (RR): LC_50_ (RQ1Ab)/LC_50_ (RQ) for the corresponding toxin. There was a significant difference if the 95% confidence limits of the LC_50_ values did not overlap.

**Table 4:** Life tables and relative fitness (mean ± SD) of the susceptible strain RQ, the Cry1Ab-resistant strain RQ1Ab, and their F_1_ progeny in *H. cunea* reared on artificial feed.

Life History Parameter	RQ (SS)	RQ1Ab (RR)	F_1_ Progeny (RS)		
			RR♂ × SS♀	SS♂ × RR♀	Pooled
Number of neonates	90 (×3)	90 (×3)	90 (×3)	90 (×3)	90 (×3)
Number of pupae	65 ± 5 ^a^	63 ± 6 ^a^	63 ± 3 ^a^	64 ± 3 ^a^	63 ± 3 ^a^
Number of adults	58 ± 3 ^a^	55 ± 3 ^a^	55 ± 3 ^a^	56 ± 3 ^a^	56 ± 2 ^a^
Number of female moths	31 ± 3 ^a^	30 ± 5 ^a^	30 ± 2 ^a^	30 ± 2 ^a^	30 ± 2 ^a^
Mean eggs laid per female	389 ± 17 ^a^	381 ± 12 ^a^	380 ± 13 ^a^	383 ± 9 ^a^	382 ± 10 ^a^
Egg viability (%)	89.6 ± 3.0 ^a^	90.0 ± 2.1 ^a^	89.9 ± 2.8 ^a^	89.4 ± 3.4 ^a^	89.6 ± 2.8 ^a^
Predicted neonate number in the next generation	10,909 ± 993 ^a^	10,420 ± 1655 ^a^	10,349 ± 516 ^a^	10,377 ± 479 ^a^	10,363 ± 445 ^a^
Net reproductive rate (R_0_)	121 ± 11 ^a^	116 ± 18 ^a^	115 ± 6 ^a^	115 ± 5 ^a^	115 ± 5 ^a^
Relative fitness ^b^	1.00 ± 0.00 ^a^	0.95 ± 0.10 ^a^	0.96 ± 0.14 ^a^	0.96 ± 0.13 ^a^	0.96 ± 0.12 ^a^

^a^ The same lowercase letter in each row means that the index does not differ significantly between different strains. ^b^ Relative fitness = R_0_ (RQ1Ab or F_1_)/R_0_ (RQ).

## Data Availability

Datasets are available on request from the authors.
